# Comparison of the Efficacy of Anatomic and Non-anatomic Hepatectomy for Hepatic Alveolar Echinococcosis: Clinical Experience of 240 Cases in a Single Center

**DOI:** 10.3389/fpubh.2021.816704

**Published:** 2022-02-08

**Authors:** Jide A, Jingni Zhang, Jinping Chai, Shunyun Zhao, Hao Wang, Xiangren A, Jinyu Yang

**Affiliations:** ^1^Medical College of Soochow University, Suzhou, China; ^2^Department of Hepatic Hydatidosis, Qinghai Provincial People's Hospital, Xining, China; ^3^Department of Internal Medicine-Cardiovascular, Qinghai Provincial People's Hospital, Xining, China; ^4^Intensive Care Unit, Qinghai Provincial People's Hospital, Xining, China; ^5^Department of Clinical Laboratory, Qinghai Province Key Laboratory of Laboratory Medicine, Qinghai Clinical Medical Research Center, Qinghai Provincial People's Hospital, Xining, China

**Keywords:** hepatic alveolar echinococcosis, non-anatomic, anatomic, hepatectomy, efficacy

## Abstract

**Background:**

Hepatic alveolar echinococcosis (AE) is a zoonotic parasitic disease. There are more than 16,000 new cases each year, approximately 60 million people are threatened, and the annual direct economic loss is RMB 3 billion. The prevalence of AE in some areas of the Qinghai–Tibet Plateau is as high as 6.0%. Radical resection, including anatomic and non-anatomic hepatectomy, for advanced AE can significantly prolong the survival time of patients. However, there is no literature compared the efficacy of anatomic and non-anatomic hepatectomy. Therefore, by comparing various clinical evaluation indices between anatomic and non-anatomic hepatectomy, this study explored the short-term and long-term efficacy of these two surgical methods for AE.

**Methods:**

The clinical data of patients with AE who underwent radical hepatectomy at Qinghai Provincial People's Hospital from January 2015 to January 2021 were retrospectively analyzed. The patients were divided into two groups by surgical method, that were, non-anatomic hepatectomy group and anatomic hepatectomy group. We compared these two groups focusing on basic preoperative data, such as age, sex, lesion size, and liver function parameters; main intraoperative evaluation indices, such as operation time, intraoperative porta hepatis occlusion time, intraoperative blood loss, and blood transfusion; and postoperative recovery evaluation indicators, such as postoperative liver function, incidence of surgical complications, and AE recurrence.

**Results:**

A total of 240 patients were enrolled in this study, including 123 in anatomic hepatectomy group and 117 in non-anatomic hepatectomy group. There were no significant differences (*P* > 0.05) between baseline characteristics. Anatomic hepatectomy group was advantageous than non-anatomic hepatectomy group regarding intraoperative blood loss (*P* < 0.001), blood transfusion (*P* < 0.001), and porta hepatis occlusion time (*P* < 0.001). There were statistically significant differences in postoperative liver function (aspartate aminotransferase: *P* < 0.001; alanine aminotransferase: *P* < 0.001), surgical complications (*P* < 0.001), and AE recurrence rate (*P* = 0.003). The median survival of patients in the anatomic hepatectomy group was 66 months, compared to 65 months in the non-anatomic hepatectomy group (χ^2^ = 4.662, *P* = 0.031).

**Conclusions:**

Anatomic hepatectomy was not only safe for AE but also showed better short-term and long-term superiority than non-anatomic hepatectomy.

## Introduction

Hepatic alveolar echinococcosis (AE) is a zoonotic parasitic disease caused by the larvae of *Echinococcus multilocularis* that seriously endangers human health (1–3). It's treatments mainly include radical resection and medication (4, 5). Medication is mainly used for early-stage AE, while radical resection is the first choice for progressive cases (6–8). The best treatment strategy is combination of surgical and postsurgical medication therapy. However, in patients whose conventional resection is not possible, other curative therapeutic options includes *ex vivo* liver resection associated with autotransplantation and liver transplantation (9). Because AE lesions are most frequently located in the right liver lobe, especially in advanced cases the major bile ducts and vessels have been invaded, major hepatic surgery is often required (10). Palliative operations have been shown to be a cause of recurrence without improving patient survival and is not recommended nowadays (11, 12).

Hepatic AE shows a similar pattern to malignancies in terms of radiologic and clinical features. For this reason, oncological surgical principles should be applied during the resection of hepatic AE. Studies have shown that based on adequate preoperative evaluation of the feasibility, and knowledge about the intraoperative techniques such as hepatic blood flow control, liver anatomy, and portal vein and biliary reconstruction, radical surgical resection can improve the quality of life and extend the survival time of the patients (13). Both anatomic hepatectomy and non-anatomic hepatectomy are radical surgical resections. However, other studies have shown that anatomic hepatectomy for AE has the advantages of less intraoperative bleeding, low incidence of postoperative complications, and rapid recovery (14). Our clinical study on anatomic hepatectomy for AE found that it has the advantages of less liver function injury, low incidence of complications, and short postoperative hospital stay (15). However, due to the shortage of early-stage cases and short follow-up times, we mainly evaluated the short-term efficacy of anatomic hepatectomy for AE. To further study, we retrospectively analyzed the clinical data of 240 patients with AE who underwent hepatectomy in Qinghai Provincial People's Hospital from January 2015 to January 2021, to explore the effect of surgical methods in the long term in patients with HAE.

## Methods

### Basic Patient Information

The clinical data of 513 patients with hepatic AE who underwent hepatectomy in Qinghai Provincial People's Hospital from January 2015 to January 2021 were retrospectively analyzed. Inclusion criteria: (1) Pathological diagnosed with AE; (2) Disease stages were I, II, or III according to the World Health Organization Informal Working Group on Echinococcosis (WHO-IWGE) PNM classification (16); (3) Without previous surgical history; (4) There was no cirrhosis, and the patient's liver function was graded as A or B before operation according to Child-Pugh classification. For the patients whose liver function was grade B, reevaluation was performed after inteventions, and if it was grade A then, they were included in the study; (5) Open hepatectomies were performed. Exclusion criteria: (1) The porta hepatis and retrohepatic inferior vena cava were severely invaded and required revascularization or *ex vivo* liver resection; (2) The surgery was palliative.

Altogether 240 patients which met above criteria were enrolled. The patients were divided into non-anatomic hepatectomy group and the anatomic hepatectomy group according to distinct surgical methods. This study was approved by the hospital ethics committee.

### Preoperative Preparation

After admission to the hospital, 240 patients underwent contrast-enhanced computed tomography and angiography (CTA) of abdomen; enzyme-linked immunosorbent assay (Diagnostic Kit for IgG Antibody to Hydatid, ELISA brand is HAI TAI and from Zhuhai special economic zone haitai biopharmaceutical Co. LTD) for the hydatid; and eight tests for infection (Including HBsAg, HBsAb, HBeAg, HBeAb, HBcAb, HCV-Ab, HIV-Ag/Ab, and TPAb). Metastasis of AE to the brain, lung, and other organs was excluded before surgery based on relevant imaging examinations. The liver function, residual liver volume, and the relationships between the lesion and the blood vessels and bile ducts were evaluated.

There were 7 patients with jaundice in this study, including 3 patients in the anatomic hepatectomy group with total bilirubin levels ranging from 54.7 to 116.4umol/L. 4 patients in the non-anatomic hepatectomy group with total bilirubin levels ranging from 44.07-154.8umol/L. However, the preoperative Child-Pugh grading of the above cases was grade B, and 3 patients underwent ultrasound-guided percutaneous liver puncture and bile duct drainage before surgery. The remaining 4 patients were treated with echinococcosis necrotic cavity puncture drainage after percutaneous liver puncture biliary drainage failed because intrahepatic bile duct dilation was not obvious. All 7 patients were treated with hepatoprotective medicine, and the liver reserve function was evaluated again after the total bilirubin level returned to normal, and the Child-pugh grade of all patients was A.

### Surgical Methods and Indications

**Figure 1:**
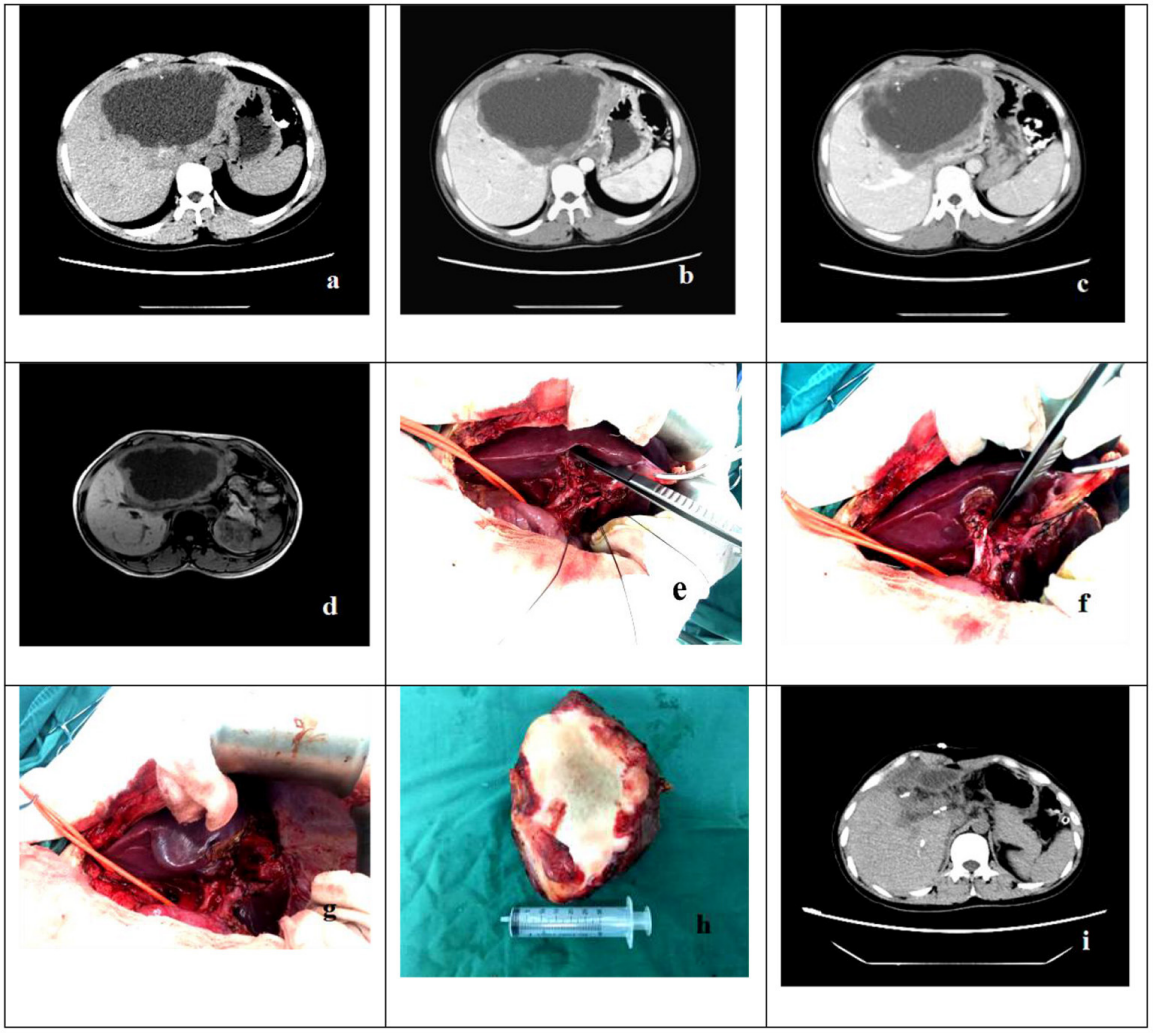
The case is a 32-year-old female patient who was hospitalized due to the chief complaint of “intermittent right upper abdominal distension, pain and discomfort for more than 4 years”. **(a–d)** Preoperative abdominal CT of patients showed that there was a lesions of echinococcosis in the liver with the size of about 12.0 × 7.0cm in S2-4 segments. **(e–h)** The intraoperative anatomy of the first hepatic portal and pathologic specimens. **(i)** Abdominal CT showed that there was residual liver compensatory and hyperplasia, but there was no recurrence of echinococcosis.

Anatomic hepatectomy: After the patient was successfully anesthetized, an inverted “L”-shaped incision was made in the upper abdomen, and the abdomen was examined layer by layer to detect whether there was metastasis of AE in the abdominal cavity. The first porta hepatis and second porta hepatis were routinely dissected and hung with ribbons to cut off the ligaments around the liver. When the resection range was large, a liver sling was placed behind the liver and in front of the vena cava. During the surgery, the central venous pressure was kept at 3–5 cmH_2_O. The blood flow into and out of the liver was selectively blocked according to the scope of resection. The range of liver ischemia was observed, and a precut line was drawn along the ischemic line and the boundary of the lesion. R0 resection was performed when the resection margin was >1.0 cm from the lesion, and R1 resection was performed along the edge of the lesion to cut it. The liver parenchyma was transected with a water jet or ultrasonic scalpel. A duct with a diameter of ≤ 0.1 cm was cauterized with an electrotome, and an intrahepatic duct with a diameter of >0.2 cm was sutured and repaired with 5-0 Prolene. The cutting surface of the liver was sutured to stop the bleeding, a drainage tube was placed in the abdominal cavity, and the abdomen was closed (Figure 1).

The indications for anatomic hepatectomy were as follows: Anatomic hepatectomy should be preferred if AE lesions are at a certain distance from the porta hepatis and retrohepatic inferior vena cava or if it would not be difficult to dissect the first porta hepatis and second porta hepatis.

**Figure 2:**
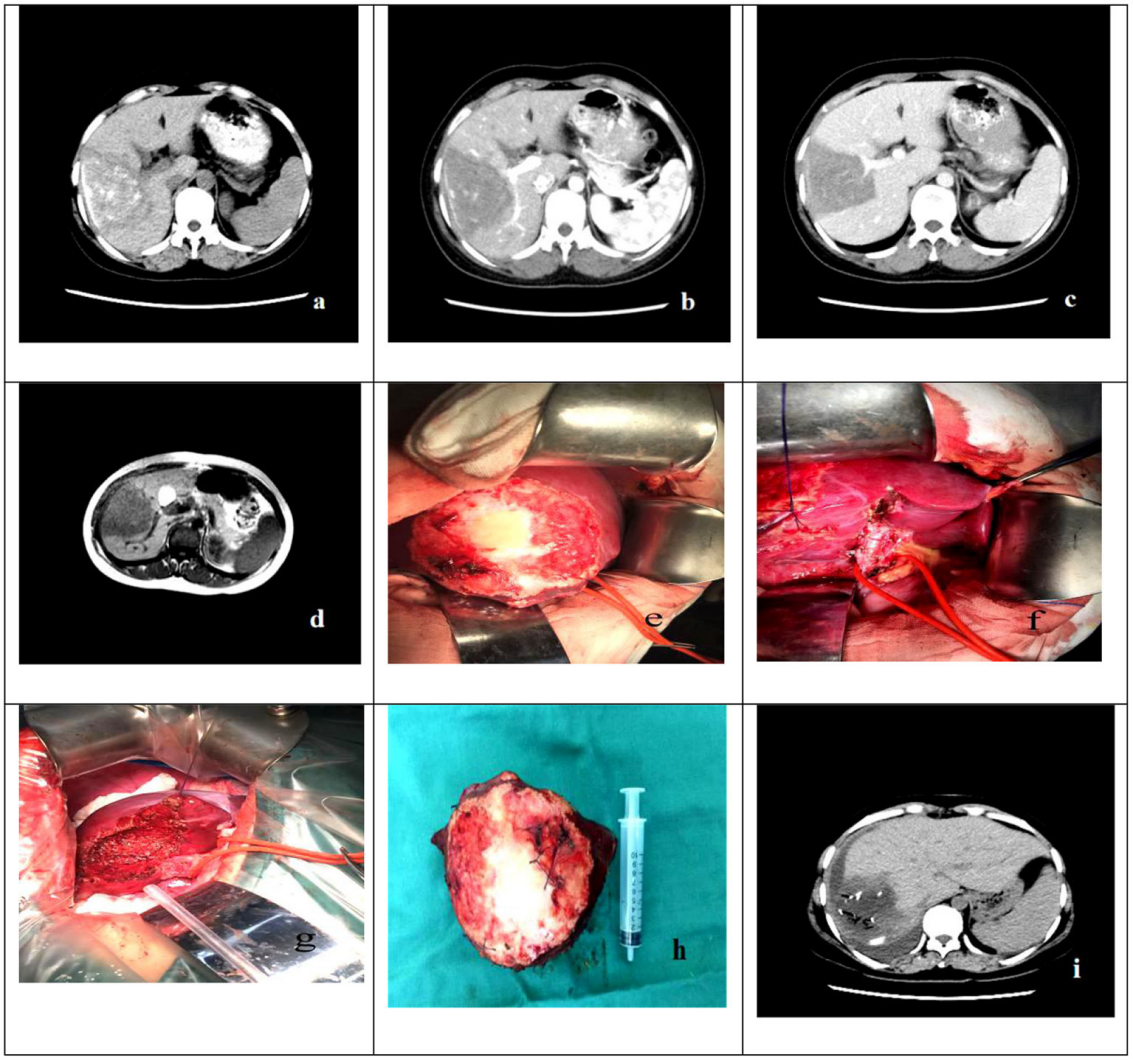
The case is a 22-year-old male patient who was hospitalized due to the chief complaint of “liver space-occupying lesions found in physical examination for more than 1 year”. **(a–d)** Preoperative abdominal CT and abdominal MRI of patient showed that there was a lesions of echinococcosis in the liver with the size of about 8.0 out.0cm in S5-6 segments. **(e–h)** The Intraoperative resection and postoperative pathological specimens. **(i)** The abdominal CT showed that there was residual liver compensatory and hyperplasia, but there was no recurrence of echinococcosis.

Non-anatomic hepatectomy: After successful general anesthesia, an inverted “L”-shaped incision was made, and the abdominal cavity was explored layer by layer. The ligaments around the liver were dissociated according to the scope of the resection, and the precut line was marked according to the size and location of the lesions. The resection margins (R0, R1) were defined as above. The routine Pringle maneuver was used to block the first porta hepatis, and the liver parenchyma was transected layer by layer along the precut line. The duct structure with a diameter of >0.2 cm was ligated with silk thread or sutured and repaired with Prolene. Smaller blood vessels and bile ducts were cauterized with an electrotome. The cutting surface of the liver was treated with gauze dipped in hot saline for hemostasis. After confirming no active bleeding, a drainage tube was placed in the abdominal cavity, and the abdomen was closed (Figure 2).

The indications for non-anatomic hepatectomy were as follows: For the surgical safety of the patient, non-anatomic hepatectomy was selected if it would be difficult to dissect the first and second porta hepatis or the AE lesions were closely associated with important ducts inside and outside the liver.

### Postoperative Management of Patients and the Administration of Albendazole

Patients in both groups were given an intravenous analgesia pump combined with subcutaneous injection of analgesics after surgery. The postoperative fluid volume was kept within 2,000–2,500 ml. The patients drank water after waking up from anesthesia and were encouraged to get out of bed as soon as possible. During the postoperative hospitalization, the patient did not take albendazole because the liver function was still recovering. Albendazole was prescribed for discharged patients in strict accordance with WHO guidelines for the diagnosis and treatment of echinococcosis (16).

### Follow-Up After Discharge

The patient was re-examined every 6 months in the first 2 years after discharge and every 12 months thereafter. The follow-up examinations mainly included ELISA for the hydatid, liver and kidney function tests, abdominal color ultrasound or abdominal CT (CT of the whole abdomen every 12 months). AE recurrence was diagnosed if imaging examination revealed new AE lesions in the liver and an ELISA for the hydatid was positive. AE recurrence was diagnosed the same way as the initial AE was, dividing it into resection margin recurrence and distant intrahepatic recurrence according to the recurrence site. Resection margin recurrence referred to recurrence when the edge of the new lesion was within 2 cm of the remaining cutting surface, and the distant intrahepatic recurrence referred to recurrence when the edge of the new lesion was >2 cm from the remaining cutting surface.

The treatments of AE recurrence, including drugs, reoperation, and comprehensive treatment, were based on the WHO guidelines for the diagnosis and treatment of echinococcosis (16). The treatment plan was chosen according to the lesion and the condition of the patient. The long-term efficacy of the patient's treatment was determined by follow-up, including over the telephone and face to face. The time between the date of surgery and the first recurrence diagnosis was defined as disease-free survival (DFS).

### Statistical Methods

SPSS 22.0 software was used for data analysis. The non-normal measurement data are represented by median and quartiles [*M*(*Q*1, *Q*3)], and they were compared between groups by the rank-sum test. Count data were analyzed by the chi-squared test with four-fold tables or the *R* × *C* chi-squared test. Repeated measurements were used to compare the trend of indicators at different time points between the two groups. The Kaplan-Meier survival curves were plotted, and the differences in survival curves were analyzed by the log-rank test. *P* < 0.05 indicates that a difference was statistically significant.

## Results

### Baseline Data

A total of 240 eligible patients with AE were enrolled, of whom 108 were males and 132 were females, with an average age of 34.20 ± 14.64 years (range: 5–79 years). Most (92.9%, 223/240) of the patients were Tibetans. Among the 240 patients, 123 underwent anatomic hepatectomy and 117 non-anatomic hepatectomy. There were no significant differences in age, sex, ethnicity, hydatid size or number, or liver function indices before the surgery between the two groups (Table 1).

**Table 1:** Comparison of baseline data between the two groups.

**Index**	**Groups**		***t/χ^2^/Z***	***P***
	**Anatomic hepatectomy**	**Non-anatomic hepatectomy**		
Age (years)	33.93 ± 16.21	34.49 ± 12.85	−0.298	0.766
Lesion size (centimeter)	11.86 ± 3.42	11.71 ± 3.74	0.339	0.735
Alanine aminotransferase before surgery (U/L)	28.33 ± 14.39	30.32 ± 15.28	−1.035	0.302
Aspartate aminotransferase before surgery (U/L)	32.31 ± 10.63	32.78 ± 14.47	−0.285	0.776
Number of hydatids	1.00 (1.00, 1.00)	1.00 (1.00, 1.00)	−0.164	0.870
Child–Pugh score	5.00 (5.00, 6.00)	5.00 (5.00, 6.00)	−0.071	0.944
**Sex**				
Male	53	55	0.372	0.542
Female	70	62		
**Ethnicity**				
Han	7	6	1.147	0.563
Tibetan	115	108		
Hui	1	3		
**Hepatitis B**				
Yes	30	31	0.140	0.708
No	93	86		
**Lesion location**				
left lobe	34	27	0.814	0.666
right lobe	77	76		
middle lobe	12	14		
**Surgical method**				
Segmental hepatectomy	69	58	3.430	0.180
Hemihepatectomy	42	30		
Extended hemihepatectomy	12	29		

*Age, hydatid size, alanine aminotransferase before surgery, aspartate aminotransferase before surgery comparison was performed using two independent samples t test. Numbe of hydatids and Child-pugh score were compared using the rank-sum test of two independent samples (Mann-Whitney U test). Sex and hepatitis B were compared by chi-squared test with four-fold tables. The R × C chi-square test was used to compare ethnicity, Lesion location and surgical method*.

### Comparison of Intraoperative and Postoperative Indices

The comparison of the intraoperative and postoperative data of the two groups showed that the time of porta hepatis occlusion, intraoperative bleeding, intraoperative blood transfusion, complication rate, and AE recurrence rate in the anatomic hepatectomy group were significantly better than those in the non-anatomic hepatectomy group. The duration of surgery was not different. The results of the surgical margins showed that the recurrence rate of R0 margins was significantly lower than that of R1 margins (χ^2^ = 175.135, *P* < 0.001, Table 2).

**Table 2:** Comparison of intraoperative and postoperative data between the two groups.

**Indices**	**Groups**		***t/χ^2^/Z***	***P***
	**Anatomic hepatectomy**	**Non-anatomic hepatectomy**		
Occlusion time (minutes)	27.36 ± 11.30	48.38 ± 20.24	−9.869	<0.001
Intraoperative bleeding (milliliter)	300.00 (200.00, 600.00)	600.00 (400.00, 1,000.00)	−6.221	<0.001
Intraoperative blood transfusion (milliliter)	0.00 (0.00, 770.00)	600.00 (0.00, 1,200.00)	−3.196	<0.001
Duration of surgery (hours)	6.24 ± 0.86	6.34 ± 0.91	−0.877	0.381
Complication				
Yes	65	104	37.394	<0.001
No	58	13		
Recurrence				
R0	5	19	9.875	0.003
sR1	118	98		

*Occlusion time and Duration of Surgery were compared using two independent sample T-tests. Two independent sample rank sum tests (Mann-Whitney U test) were used in the comparison of intraoperative bleeding and Intraoperative blood transfusion. The chi-squared test with four-fold tables was adopted for comparison between complication and recurrence*.

### Comparison of Liver Function Indices at Different Times

**Figure 3:**
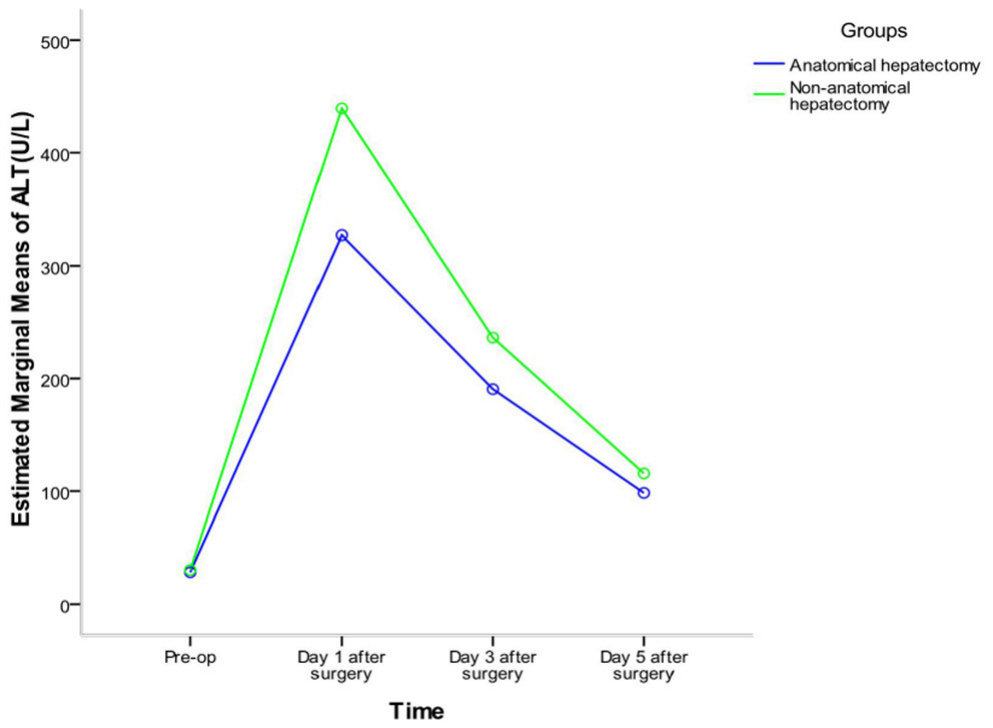
Interactive profile of time factor and grouping factor.

**Figure 4:**
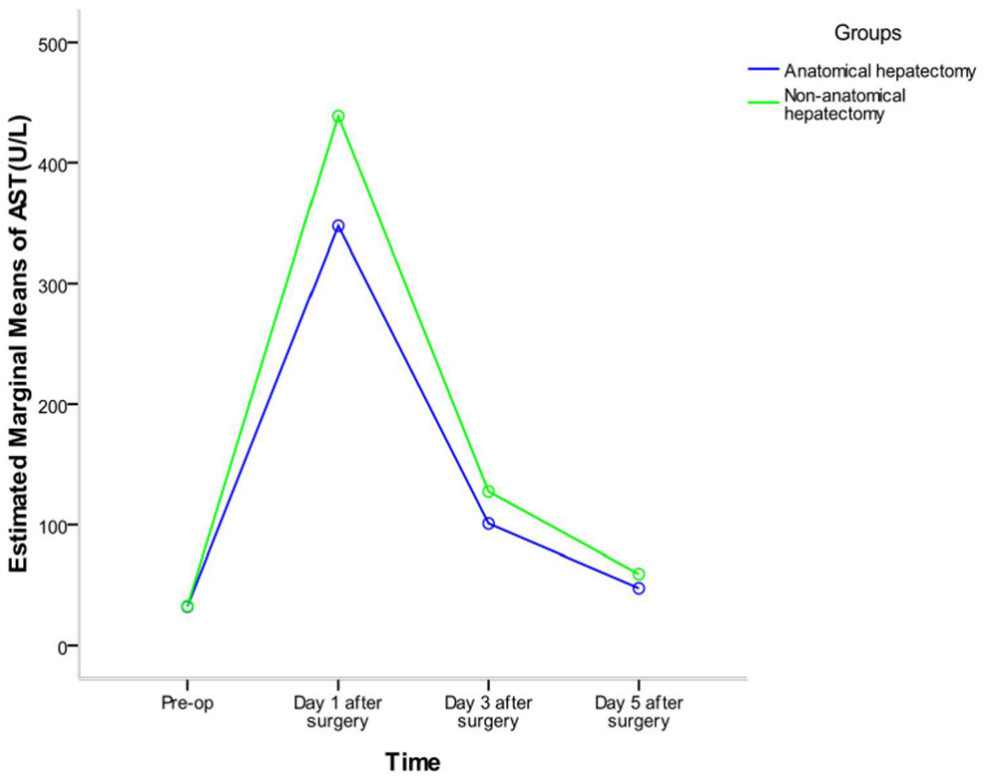
Interactive profile of time factor and grouping factor.

Repeated-measures analysis of variance of alanine aminotransferase (ALT) and aspartate aminotransferase (AST) of the two groups at different times showed that there were significant differences in ALT before and after surgery in the whole sample (*F* = 411.767, *P* < 0.001) and in each group (anatomic hepatectomy group: *F* = 274.307, *P* < 0.001; non-anatomic hepatectomy group: *F* = 182.380, *P* < 0.001). The trend of ALT in the two groups was the same: ALT was the lowest before surgery, peaked on the first day after surgery, and decreased gradually on the third and fifth days after surgery. The concentration of ALT in the anatomic hepatectomy group was significantly lower than that in the non-anatomic hepatectomy group (*F* = 17.022, *P* <0.001). There was no significant difference in ALT between the two groups before surgery, but it was significantly lower in the anatomic hepatectomy group at all other time points. There was an interaction effect between ALT expression time and surgical method (*F* = 10.475, *P* < 0.001). The highest ALT expression was 1 day after surgery in the non-anatomic hepatectomy group, and the lowest was before surgery in the anatomic hepatectomy group. The changes in AST concentration in both groups were the same as those of ALT (Table 3 and Figures 3, 4).

**Table 3:** Comparison of liver function indices between the two groups at different times before and after surgery.

**Indices**	**Time**				**Sum**	***F***	***P***
	**Before surgery**	**1 day after surgery**	**3 days after surgery**	**5 days after surgery**			
**ALT (U/L)**							
Anatomic hepatectomy	28.33 ± 14.39	327.02 ± 169.10	190.52 ± 77.81	98.35 ± 52.08	161.06 ± 147.82	274.307	<0.001
Non-anatomic hepatectomy	30.32 ± 15.28	439.46 ± 280.63	236.32 ± 135.86	115.64 ± 64.27	205.43 ± 221.12	182.380	<0.001
Sum	29.30 ± 14.83	381.84 ± 236.63	212.85 ± 112.14	106.78 ± 58.86	182.69 ± 188.39	411.767^*^	<0.001^*^
*t*	−1.035	−3.736	−3.183	−2.283	17.022^*^	10.475^#^	<0.001^#^
*P*	0.302	<0.001	0.002	0.023	<0.001^*^		
**AST (U/L)**							
Anatomic hepatectomy	32.31 ± 10.63	347.67 ± 173.31	100.91 ± 60.75	47.50 ± 16.96	132.10 ± 157.01	335.436	<0.001
Non-anatomic hepatectomy	32.78 ± 14.47	438.70 ± 255.10	127.46 ± 52.49	59.16 ± 32.61	164.77 ± 208.66	249.69	<0.001
Sum	32.54 ± 12.62	392.05 ± 221.35	113.85 ± 58.29	53.19 ± 26.40	148.01 ± 184.61	554.871^*^	<0.001^*^
*t*	−0.285	−3.217	−3.615	−3.448	16.747^*^	8.170^#^	0.003^#^
*P*	0.776	0.002	<0.001	0.001	<0.001^*^		

* *indicates the F-statistic and P-value of the main effect*;

# *indicates the F-statistic and P-value of the interaction*.

*Two independent sample t-test was used to compare ALT and AST between different groups at the same time, and one-way repeated measurement analysis of variance was used to compare the changes of ALT and AST between the same group at different times*.

### Comparison of Survival Time Between the Two Groups

**Figure 5:**
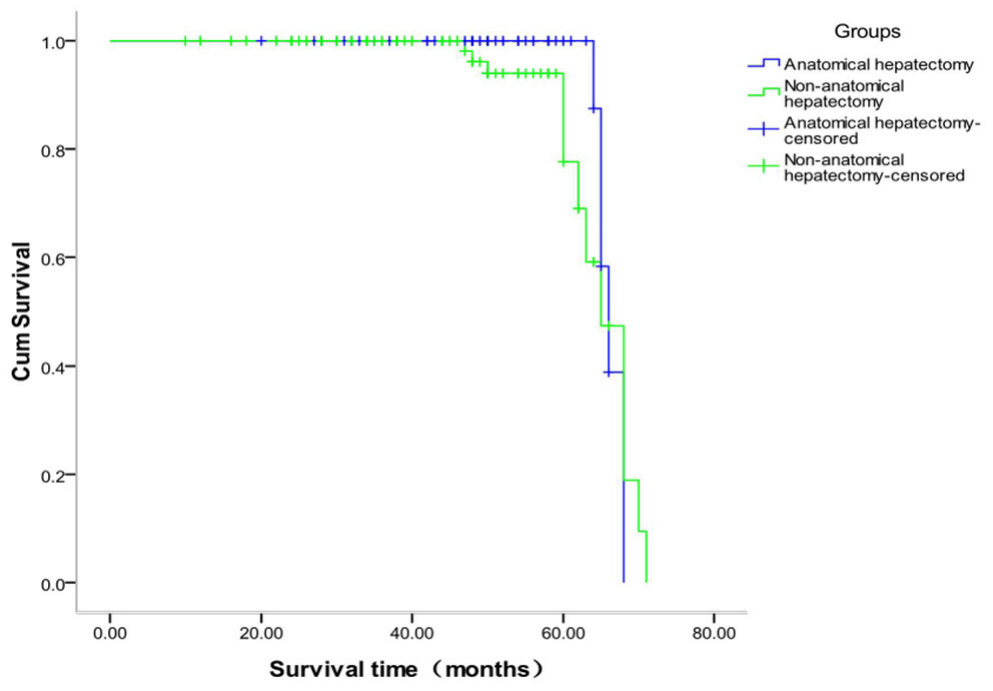
Comparison of survival curves between the two groups with different surgical methods.

**Figure 6:**
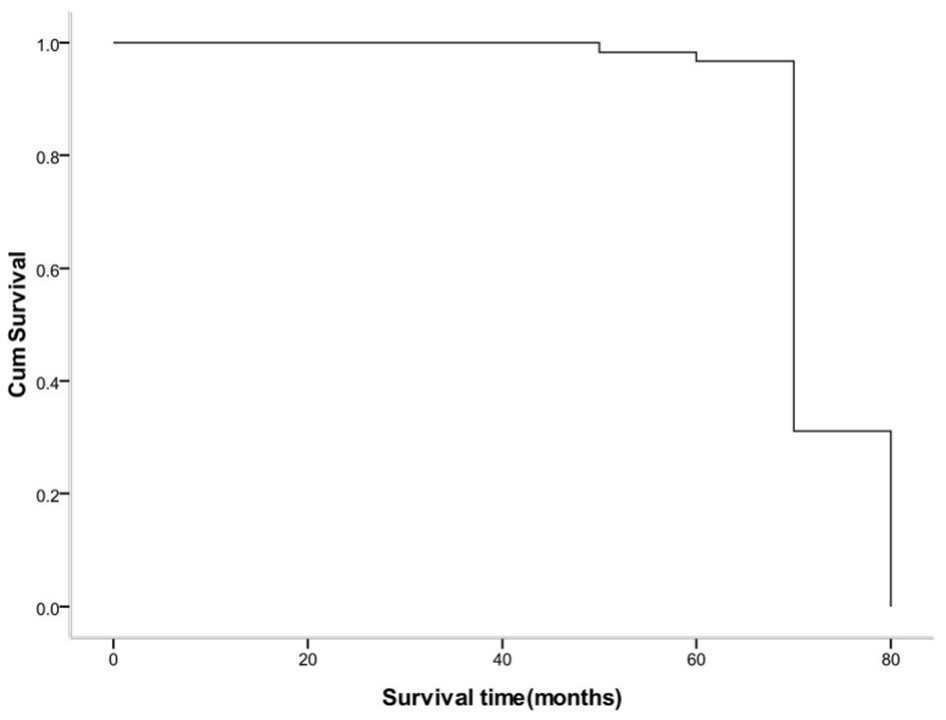
Cumulative survival curve of hydatid patients.

The survival analysis of the two groups showed that 118 cases were censored in the anatomic hepatectomy group, for a censoring rate of 95.9%, and 99 cases in the non-anatomic hepatectomy group, for a censoring rate of 84.6%. The median survival time of patients in the anatomic hepatectomy group was 66 months, compared to 65 months in the non-anatomic hepatectomy group (χ^2^ = 4.662, *P* = 0.03, Figure 5). From the survival curve in Figure 3, the prognosis of patients in the anatomic hepatectomy group was better than that in the non-anatomic hepatectomy group. The survival analysis of all patients showed that the median survival time of patients was 67.12 months, as shown in Figure 6.

### Survival Time Analysis of Different Margins in the Anatomic and Non-anatomic Groups

**Figure 7:**
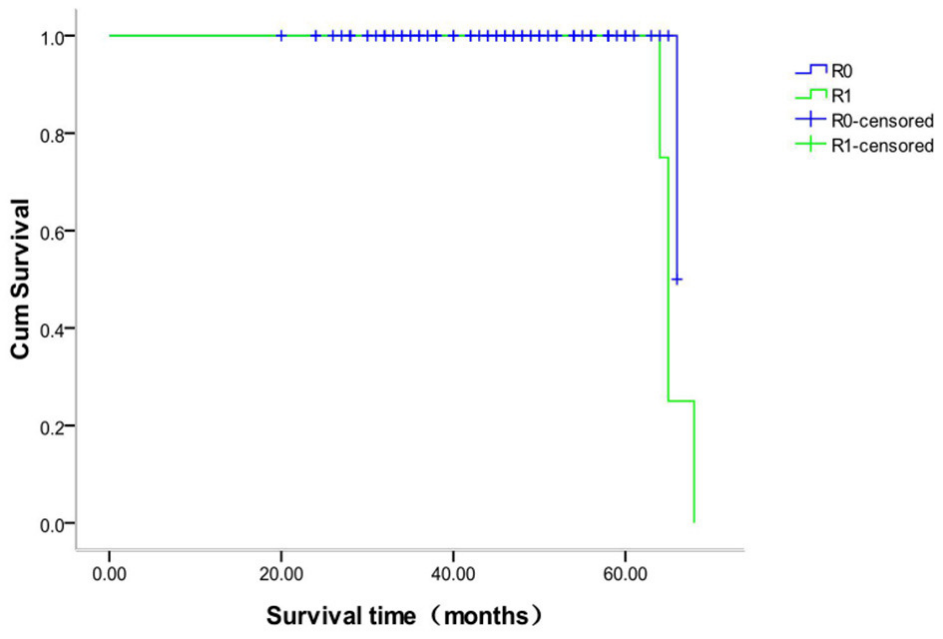
Survival curve of patients with different surgical margins after anatomical hepatectomy.

**Figure 8:**
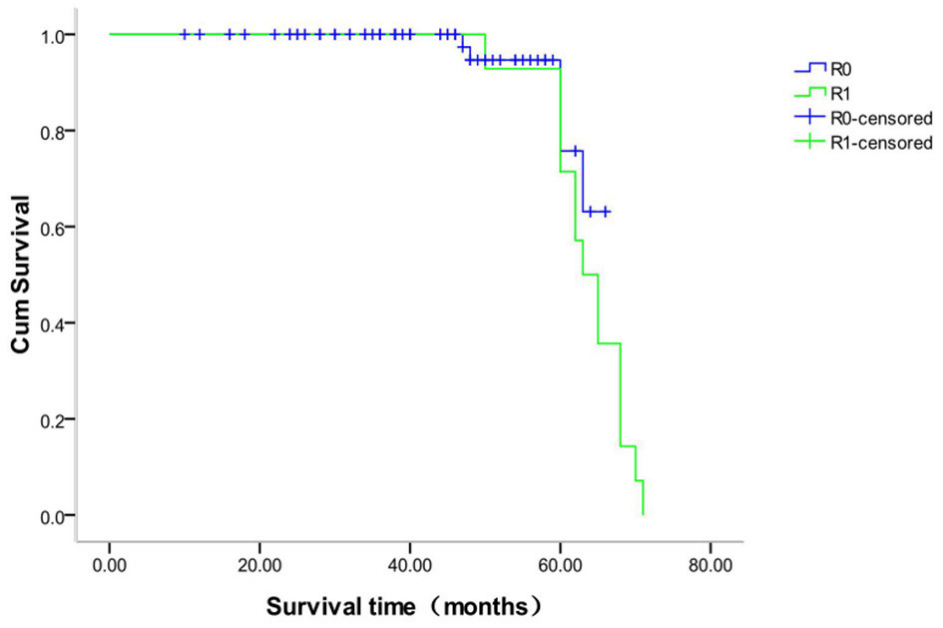
Survival curve of patients with different surgical margins after non-anatomical hepatectomy.

The survival analysis of patients with different margins in the anatomic hepatectomy group showed that 118 patients with R0 margin were censored, for a censoring rate of 99.2%, and no patient was censored with an R1 margin. The median survival time of patients with R0 margins was 66 months and R1 margins 65 months (χ^2^ = 1.561, *P* = 0.212, Figure 7). The survival analysis of patients with different margins in the non-anatomic hepatectomy group showed that 98 patients with R0 margin were censored, for a censoring rate of 95.1%, and no patient was censored with R1 margins. There was no significant difference in the survival time between these two sub-groups (χ^2^ = 0.947, *P* = 0.330, Figure 8).

## Discussion

Hepatic AE is called “hydatid cancer” due to its special biological characteristics (17, 18). Radical resection of lesions is an effective treatment for advanced hepatic AE (19, 20). Anatomic hepatectomy is not only the basic method of precision liver surgery but also the ideal method of liver tumor resection (21, 22). Studies in China (23, 24) have shown that anatomic hepatectomy is safe and reliable for hepatic AE, with the advantages of fewer surgical complications and rapid recovery. The cited studies have mainly evaluated the short-term efficacy of anatomic hepatectomy for hepatic AE, with a postoperative follow-up time <1 year. Therefore, in this study, 240 patients with hepatic AE after hepatectomy were followed up for a long time, and the follow-up data were statistically processed to better evaluate the long-term efficacy of anatomic hepatectomy for hepatic AE.

This study mainly focused on the following three results. First, the short-term efficacy of anatomic hepatectomy was significantly better than that of non-anatomic hepatectomy in terms of rapid recovery of liver function and a low incidence of complications. Anatomic hepatectomy can best maintain the integrity of the residual liver structure and function by precise intraoperative methods, selective hepatic blood flow occlusion, and low central venous pressure, and these measures can effectively control intraoperative blood loss, since intraoperative blood loss and blood transfusion are closely correlated with poor outcomes (25). At the same time, selective hepatic blood flow occlusion can effectively reduce the ischemia–reperfusion injury of residual liver tissue (26–29). However, the focus of this clinical study was the long-term efficacy of anatomic hepatectomy for hepatic AE. Second, there was no difference in the overall survival time between the anatomic and non-anatomic hepatectomy groups, but the DFS time of patients in the anatomic hepatectomy group was significantly longer than that in the non-anatomic hepatectomy group. Similar results have been reported in studies of the prognosis of liver cancer (30, 31). Studies abroad have shown that radical resection for hepatic AE can significantly prolong the DFS of patients (6, 7). Studies in China (32, 33) have shown that some patients with hepatic AE have a high recurrence rate even after radical resection of lesions and regular oral administration of anti-echinococcosis drugs after operation. The main reason may be related to the range of surgical resection. Wen et al. (34) showed that the main factor for hepatic AE recurrence was the control of surgical margins. Shabunin et al. (35) reported more than 2 cm of that normal liver tissue around the lesion should be removed during radical resection for AE in order to reduce the postoperative recurrence rate. For this reason, the academic community in China has reached a consensus that during the thorough removal of echinococcosis lesions, the normal liver tissue more than 1 cm away from the lesion edge should be removed, aiming to eliminate the “infiltration zone” with active hyperplasia around lesions and reduce postoperative recurrence (36, 37). The infiltration zone is the location of actively proliferating cells, which is rich in microvessels and mainly includes the portal vein and hepatic artery. AE lesions are constantly infiltrating and growing into lesion microenvironment, which concept was firstly established by Dr. Aini et al. (38–40). This study found that the infiltration zone had not only active hyperplasia but also microvascular invasion of AE, which is similar to the microvascular invasion in the tissues adjacent to liver cancer. Microvascular invasion is closely related to the prognosis of liver cells (41, 42), but whether microvascular invasion of AE is related to postoperative recurrence is not known. Finally, in clinical practice, we often encounter irregular AE lesions, or lesions adjacent to the porta hepatis or retrohepatic inferior vena cava. In such cases, sufficient margin width (>1 cm) cannot be achieved, and only complete resection of the lesions and negative margins can be achieved. However, a recent research that studied AE lesion microenvironment proposed that different lesion categories had different infiltrative boundary, thus, tailored resection margin was strongly recommended (40). Therefore, any resection margin that do not consider lesion heterogeneity would not be appropriative in the era of precision management. These shortcomings may explain the high recurrence rate of patients in the non-anatomic hepatectomy group. Joliat also showed that when radical resection for AE was performed, the recurrence rate of patients with positive margins confirmed by postoperative pathology was as high as 41% at 7 years, even with the postoperative adjuvant albendazole treatment (43).

## Conclusion

In conclusion, for hepatic AE, anatomic hepatectomy can achieve good long-term efficacy only on the premise of ensuring a large enough resection range. In addition to comparing the efficacy of the two surgical methods, this study examined the factors related to postoperative recurrence of hepatic AE, and we will continue to study this topic.

## Data Availability Statement

The original contributions presented in the study are included in the article/supplementary material, further inquiries can be directed to the corresponding authors.

## Ethics Statement

The studies involving human participants were reviewed and approved by the Ethics Committee of the Qinghai Provincial People's Hospital. Written informed consent to participate in this study was provided by the participants' legal guardian/next of kin.

## Author Contributions

JA, SZ, and HW: conceived and designed the study. JA, JZ, JC, and XA: collected the data. JA and JZ: contributed to data analysis and interpretation. JA and JC: writing article. JY and XA: approved the study and this submission. All authors contributed to the article and approved the submitted version.

## Funding

This work was supported by the Basic Research Project of Qinghai Province (Nos: 2020-wjzdx-27, 2022-0301-ZJC-0114, and 2022-0301-ZJC-0051), Qinghai Province Key Laboratory of Laboratory Medicine, Qinghai Clinical Medical Research Center (No: 2018-SF-L3).

## Conflict of Interest

The authors declare that the research was conducted in the absence of any commercial or financial relationships that could be construed as a potential conflict of interest.

## Publisher's Note

All claims expressed in this article are solely those of the authors and do not necessarily represent those of their affiliated organizations, or those of the publisher, the editors and the reviewers. Any product that may be evaluated in this article, or claim that may be made by its manufacturer, is not guaranteed or endorsed by the publisher.
