# Corrigendum: Comparison of the Efficacy of Anatomic and Non-anatomic Hepatectomy for Hepatic Alveolar Echinococcosis: Clinical Experience of 240 Cases in a Single Center

**DOI:** 10.3389/fpubh.2022.917531

**Published:** 2022-05-16

**Authors:** Jide A, Jingni Zhang, Jinping Chai, Shunyun Zhao, Hao Wang, Xiangren A, Jinyu Yang

**Affiliations:** ^1^Medical College of Soochow University, Suzhou, China; ^2^Department of Hepatic Hydatidosis, Qinghai Provincial People's Hospital, Xining, China; ^3^Department of Internal Medicine-Cardiovascular, Qinghai Provincial People's Hospital, Xining, China; ^4^Intensive Care Unit, Qinghai Provincial People's Hospital, Xining, China; ^5^Department of Clinical Laboratory, Qinghai Province Key Laboratory of Laboratory Medicine, Qinghai Clinical Medical Research Center, Qinghai Provincial People's Hospital, Xining, China

**Keywords:** hepatic alveolar echinococcosis, non-anatomic, anatomic, hepatectomy, efficacy

In the published article, there was an error in affiliation “1”. Instead of “Soochow University, Suzhou, China,” it should be “Medical College of Soochow University, Suzhou, China.”

The authors apologize for this error and state that this does not change the scientific conclusions of the article in any way. The original article has been updated.

## Publisher's Note

All claims expressed in this article are solely those of the authors and do not necessarily represent those of their affiliated organizations, or those of the publisher, the editors and the reviewers. Any product that may be evaluated in this article, or claim that may be made by its manufacturer, is not guaranteed or endorsed by the publisher.

